# p27 controls autophagic vesicle trafficking in glucose-deprived cells via the regulation of ATAT1-mediated microtubule acetylation

**DOI:** 10.1038/s41419-021-03759-9

**Published:** 2021-05-13

**Authors:** Ada Nowosad, Justine Creff, Pauline Jeannot, Raphael Culerrier, Patrice Codogno, Stephane Manenti, Laurent Nguyen, Arnaud Besson

**Affiliations:** 1 MCD, Centre de Biologie Intégrative, Université de Toulouse, CNRS, UPS, 31062 Toulouse, Cedex France; 2 Institut Necker-Enfants Malades (INEM), INSERM U1151-CNRS UMR 8253, F-75015 Paris, France; 3 Université Paris, F-75005 Paris, France; 4 Cancer Research Center of Toulouse (CRCT), INSERM U1037, CNRS ERL5294, University of Toulouse, Toulouse, France; 5 GIGA-Stem Cells, University of Liège, CHU Sart Tilman, Liège, Belgium

**Keywords:** Tumour-suppressor proteins, Macroautophagy

## Abstract

The cyclin-dependent kinase inhibitor p27^Kip1^ (p27) has been involved in promoting autophagy and survival in conditions of metabolic stress. While the signaling cascade upstream of p27 leading to its cytoplasmic localization and autophagy induction has been extensively studied, how p27 stimulates the autophagic process remains unclear. Here, we investigated the mechanism by which p27 promotes autophagy upon glucose deprivation. Mouse embryo fibroblasts (MEFs) lacking p27 exhibit a decreased autophagy flux compared to wild-type cells and this is correlated with an abnormal distribution of autophagosomes. Indeed, while autophagosomes are mainly located in the perinuclear area in wild-type cells, they are distributed throughout the cytoplasm in p27-null MEFs. Autophagosome trafficking towards the perinuclear area, where most lysosomes reside, is critical for autophagosome–lysosome fusion and cargo degradation. Vesicle trafficking is mediated by motor proteins, themselves recruited preferentially to acetylated microtubules, and autophagy flux is directly correlated to microtubule acetylation levels. p27^−/−^ MEFs exhibit a marked reduction in microtubule acetylation levels and restoring microtubule acetylation in these cells, either by re-expressing p27 or with deacetylase inhibitors, restores perinuclear positioning of autophagosomes and autophagy flux. Finally, we find that p27 promotes microtubule acetylation by binding to and stabilizing α-tubulin acetyltransferase (ATAT1) in glucose-deprived cells. ATAT1 knockdown results in random distribution of autophagosomes in p27^+/+^ MEFs and impaired autophagy flux, similar to that observed in p27^−/−^ cells. Overall, in response to glucose starvation, p27 promotes autophagy by facilitating autophagosome trafficking along microtubule tracks by maintaining elevated microtubule acetylation via an ATAT1-dependent mechanism.

## Introduction

p27^Kip1^ (p27) is a cell cycle inhibitor that restrains cell proliferation by binding to cyclin/CDK complexes and inhibiting their activity^1^. In this way, p27 acts as a tumor suppressor and loss of p27 expression in mice promotes tumor development^2,3^. In many cancers, oncogenic kinases phosphorylate p27, leading to its cytoplasmic localization, which inactivates its tumor suppressor functions^4–7^. Increasing evidence indicates that cytoplasmic localization of p27 confers resistance to anticancer therapies and negatively affects cancer patient prognosis^7,8^. p27 may sustain cancer progression via several mechanisms, including the regulation of cell migration and invasion^9–12^, stemness^13,14^, and autophagy^15–21^.

Macroautophagy (hereafter autophagy) is a physiological self-digestion process in which intracellular components are sequestrated within double-membrane autophagosomes and delivered to lysosomes for degradation^22^. In normal conditions, autophagy occurs at basal levels to keep cells healthy by preventing the accumulation of damaged organelles and proteins. During stress, such as starvation, hypoxia, ionizing radiations, or chemotherapeutic treatment, autophagy levels dramatically increase, which is considered as a self-defense mechanism^22^. In the context of cancer, autophagy is thought to initially prevent tumor development by participating in the maintenance of cytoplasm quality and genome integrity. In contrast, in established tumors, autophagy sustains proliferation and survival by generating energy and substrates for anabolic reactions^23^. Upon energetic stress, 5’-AMP-activated protein kinase (AMPK) phosphorylates the ATG1/ULK1 kinase to initiate autophagy. Then, a subset of ATG (autophagy-related) proteins is recruited to the site of autophagosome formation on the endoplasmic reticulum membrane. ATG proteins orchestrate all steps of autophagy and are used as markers to monitor this process. Transcriptional regulation and post-translational modifications of ATG proteins play a crucial role in the control of autophagy^24^. Additionally, other factors such as cytoskeleton and membrane dynamics regulate autophagy under basal and stress conditions^25^. Membranes play an important role in the early steps of autophagy as a source of lipid for expanding autophagosomes. On the other hand, the actin, intermediate filament, and microtubule (MT) cytoskeleton have all been reported to contribute to both early and late stages of autophagy^26^.

During autophagosome formation, MT act as a scaffold for the assembly of the autophagy machinery and for signaling proteins that regulate the extent of the autophagic response^25,26^. Once formed, autophagosomes are transported along MT tracks towards the minus-end of MTs and the microtubule-organizing center (MTOC), where most lysosomes reside^26–28^. This retrograde transport is mediated by dyneins^27,28^. However, kinesin-dependent plus-end movement also appears to be needed, as autophagosomes accumulate and fail to fuse with lysosomes in KIF5B-depleted cells^29^. This is in agreement with other reports indicating that autophagosomes move bidirectionally along MTs to finally accumulate in the vicinity of the MTOC^30^. MT acetylation was shown to promote the recruitment of motor proteins to MT and intracellular trafficking^25,31,32^, as well as autophagosome–lysosome fusion and autophagy^33–36^. Nevertheless, how MT acetylation is controlled during autophagy is still unclear.

p27 was recently implicated in the promotion of basal and stress-induced autophagy^15–21^. Following glucose and/or serum deprivation, LKB1 activates AMPK, which in turn phosphorylates p27 on T198, T170 and/or S83, causing its cytoplasmic retention and promoting autophagy^17,37^. Expression of a p27 mutant localizing in the cytoplasm was sufficient to promote autophagy^17^. Upon glucose deprivation, p27-mediated autophagy confers resistance to apoptosis, suggesting that p27 plays an important role in the cellular response to energetic stress^17^. While the pro-autophagic role of p27 is well established, little is known about the molecular mechanism by which p27 favors autophagy upon starvation.

Here, we investigated how p27 promotes autophagy in response to glucose starvation. Our data indicate that cytoplasmic p27 facilitates autophagy by promoting the transport of autophagosomes towards the MTOC and their fusion with lysosomes. At the molecular level, p27 promotes MT acetylation by binding to and stabilizing ATAT1, an enzyme responsible for MT acetylation. Glucose-deprived p27 knockout cells exhibit an autophagosome positioning defect, impaired autophagy flux, and low MT acetylation levels due to reduced ATAT1 expression. Increasing MT acetylation by treatment with HDAC inhibitors or re-expression of p27 rescues the autophagy defect of p27-null MEFs. Taken together, these data reveal the molecular mechanism by which p27 promotes autophagy in response to glucose starvation.

## Results

### p27 promotes autophagy during glucose deprivation

**Fig. 1:**
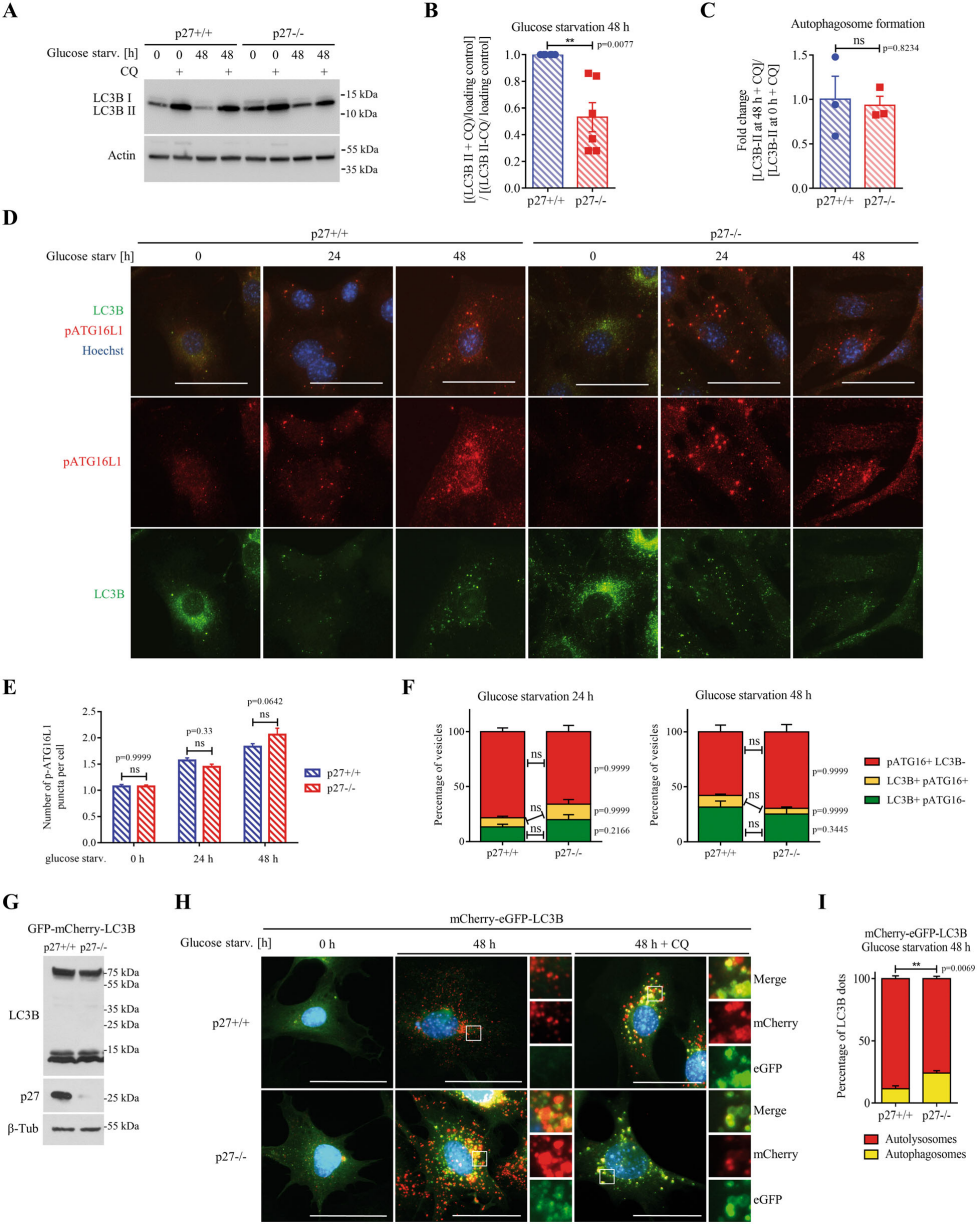
p27 promotes autophagy flux in glucose-deprived cells. **A** LC3B immunoblot in p27^+/+^ and p27^−/−^ MEFs in full medium (0 h) or glucose-deprived for 48 h ± CQ (50 µM) for 2 h before collecting cells. Actin was used as loading control. **B** LC3 turnover, measured as a ratio of LC3B-II signal intensity in starved cells in the presence of CQ versus LC3B-II under the same conditions without CQ, as described in A. Graph shows means ± SEM from *n* = 6 independent experiments. **C** Autophagosome formation was evaluated by measuring the ratio of LC3B-II levels, in the presence of CQ to block lysosomal degradation, at 48 h and 0 h of glucose starvation. Graph shows means ± SEM from *n* = 3 independent experiments. **D** p27^+/+^ and p27^−/−^ MEFs were glucose-starved for the indicated times and stained for p-ATG16L1 and LC3B. DNA was stained with Hoechst 33342. **E** Graph shows the average number of p-ATG16L1 puncta per cell ± SEM. Number of cells used for quantification: p27^+/+^: *n* = 210 (0 h), 625 (24 h), *n* = 822 (48 h); p27^−/−^: *n* = 222 (0 h), *n* = 906 (24 h), *n* = 284 cells (48 h). **F** Graphs show mean ± SEM of the distribution of autophagophores (p-ATG16L1+/LC3B−, red), early autophagosomes (p-ATG16L1+/LC3B+, yellow) and mature/sealed autophagosomes (p-ATG16L1−/LC3B+, green) in cells deprived of glucose for 24 h (p27^+/+^: *n* = 15; p27^−/−^: *n* = 27 images and 48 h (p27^+/+^: *n* = 16; p27^−/−^: *n* = 19 images). **G** Immunoblot for LC3B and p27 in mCherry-eGFP-LC3B MEFs used in experiment described in (**H**). In the LC3B immunoblot, the 75 kDa band is mCherry-GFP-LC3B and the ~13 kDa doublet is endogenous LC3B. β-tubulin was used as loading control. **H** p27^+/+^ and p27^−/−^ MEFs expressing mCherry-eGFP-LC3B were glucose deprived for 48 h and fixed prior to microscopy analysis. CQ treatment for 2 h was used as negative control. Scale bars are 50 µm. **I** Quantification of experiments as described in (**H**) in MEFs glucose starved for 48 h. Yellow LC3B dots represent autophagosomes, while red LC3B dots represent autolysosomes. Graph shows mean ± SEM number of autolysosomes and autophagosomes from *n* = 3 independent experiments. At least 150 LC3B dots were analyzed per conditions per experiment. **B**, **C**, **E**, **F**, **I** Statistical significance was evaluated by two-tailed Student’s *t* test with Welch’s correction (B, C) or 2-way ANOVA (**E**, **F**, **I**).

p27 was previously reported to promote autophagy and cell survival in response to glucose and/or serum deprivation^17^. To investigate the mechanism underlying the role of p27 in autophagy, p27^+/+^ and p27^−/−^ mouse embryo fibroblasts (MEFs) were glucose-deprived and autophagy was evaluated by monitoring ATG8/MAP1LC3/LC3B (hereafter LC3B) levels. Since autophagy is a highly dynamic process and LC3B undergoes rounds of synthesis and degradation, LC3B-II levels were measured in the presence and absence of chloroquine (CQ), which blocks lysosomal degradation, to determine autophagy flux, represented by the ratio between LC3B-II levels in the presence and absence of CQ^38^. Upon glucose deprivation, autophagic flux was significantly lower in p27^−/−^ MEFs compared to wild-type cells (Fig. 1A, B). The overall expression of LC3B may also be used as a marker of autophagy flux^38^. Monitoring of turboGFP (tGFP) fluorescence signal in cells expressing tGFP-LC3B with an IncuCyte showed an accumulation of LC3B signal in p27^−/−^ MEFs during glucose deprivation (Fig. S1A, B), suggesting reduced LC3B turnover and impaired autophagy flux. p62 is known as a selective substrate of autophagy and as such, its accumulation during starvation is a good indicator of impaired autophagy flux^38^. After 48 h of glucose starvation, p62 levels, monitored by immunofluorescence (Fig. S1C, D) and immunoblot (Fig. S1E, F), were lower in p27^+/+^ MEFs compared to p27^−/−^ cells, supporting the idea that p27 promotes autophagy and that autophagy flux is partially impaired in the absence of p27.

A series of experiments was then performed to determine which step of the autophagic process is affected by p27. First, autophagosome formation was assessed by measuring LC3B-II levels at 0 h and 48 h of glucose starvation in the presence of CQ to block lysosomal proteolysis^39^ and similar levels were detected in p27^+/+^ and p27^−/−^ MEFs (Fig. 1C), indicating that p27 does not affect autophagosome biogenesis governed by ATG proteins. This was confirmed by monitoring ATG16L1 phosphorylation on S278 and LC3B signal that allows distinguishing between newly formed autophagophores (p-ATG16L1+, LC3B−), expanding autophagosomes (p-ATG16L1+, LC3B+) and sealed/mature autophagosomes (p-ATG16L1-, LC3B+), which did not show any significant difference between p27^+/+^ and p27^−/−^ cells (Fig. 1D–F)^40^. Similarly, the knockout of p27 did not affect the number and nature of ATG5-positive vesicles (Fig. S1A–C), which represent early autophagic structures^41,42^, suggesting that p27 is dispensable for early autophagy events. To distinguish between autophagosomes and autolysosomes, we used MEFs stably expressing tandem fluorescent mCherry-eGFP-LC3B (Fig. 1G), in which autophagosomes appear as yellow puncta due to mCherry and GFP fluorescence, while autolysosomes appear in red as a result of GFP fluorescence quenching in the acidic environment of the lysosomal lumen^43^. Upon glucose deprivation, p27^−/−^ MEFs exhibited less red autolysosome puncta than p27^+/+^ cells, suggesting defective autophagosome maturation in the absence of p27 (Fig. 1H, I).

**Fig. 2:**
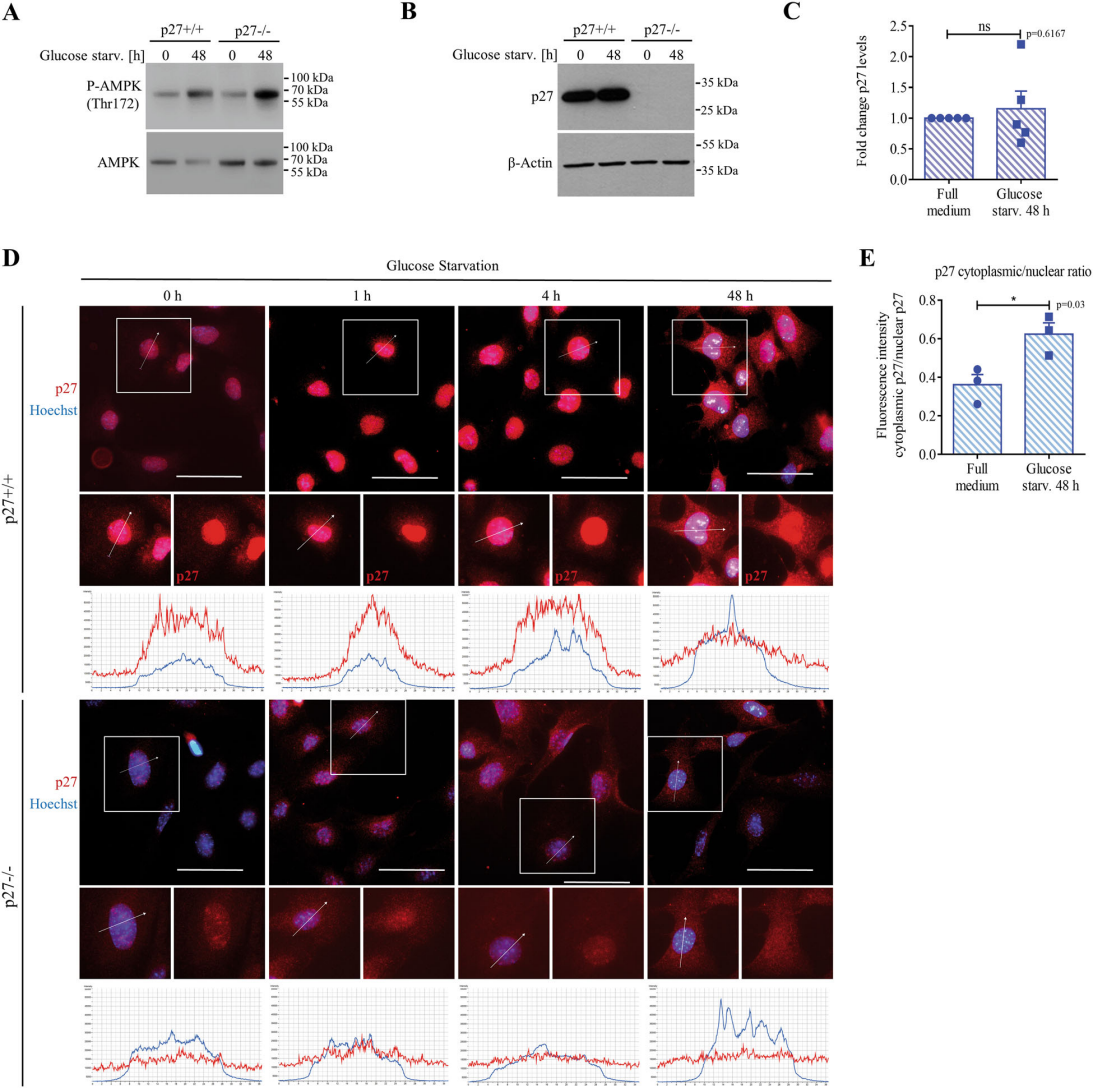
p27 localizes in the cytoplasm during prolonged glucose starvation. **A** Immunoblot for P-T172-AMPK and AMPK of p27^+/+^ and p27^−/−^ MEFs in full medium (0 h) or glucose-starved for 48 h. **B** p27 immunoblot of p27^+/+^ and p27^−/−^ MEFs in full medium (0 h) or glucose-starved for 48 h. β-actin was used as loading control. **C** Graph shows mean ± SEM of quantification of p27 levels from experiments shown in B, expressed as fold change from full medium condition from *n* = 5 independent experiments. **D** p27^+/+^ and p27^−/−^ MEFs were glucose-deprived for the indicated times and immunostained for p27. Nuclear DNA was stained with Hoechst 33342. Graphs display the fluorescence intensity (arbitrary unit) in each channel over the distance depicted by the arrows. **E** Graph shows means ± SEM of the cytoplasm to nuclear ratio of p27 fluorescence intensity in p27^+/+^ and p27^−/−^ MEFs in full medium (0 h) or glucose-starved for 48 h from *n* = 3 independent experiments. At least 64 cells were analyzed per condition per experiment. **C**, **E** Statistical significance was evaluated by two-tailed Student’s *t* test with Welch’s correction.

Glucose deprivation leads to AMPK activation and the subsequent phosphorylation of p27, causing its cytoplasmic retention and stabilization^17,20,37,44^. In agreement with these reports, AMPK was activated in glucose-deprived MEFs (Fig. 2A) and p27 levels slightly increased, although not significantly (Fig. 2B, C). Similarly, monitoring p27 subcellular localization by immunofluorescence showed a progressive relocalization of p27 in the cytoplasm and an increase in the cytoplasmic to nuclear ratio of p27 after 48 h of glucose starvation (Fig. 2D, E). Thus, prolonged glucose deprivation induces p27 relocalization to the cytoplasm, which promotes autophagy.

### p27 regulates autophagosome positioning in glucose-deprived cells

**Fig. 3:**
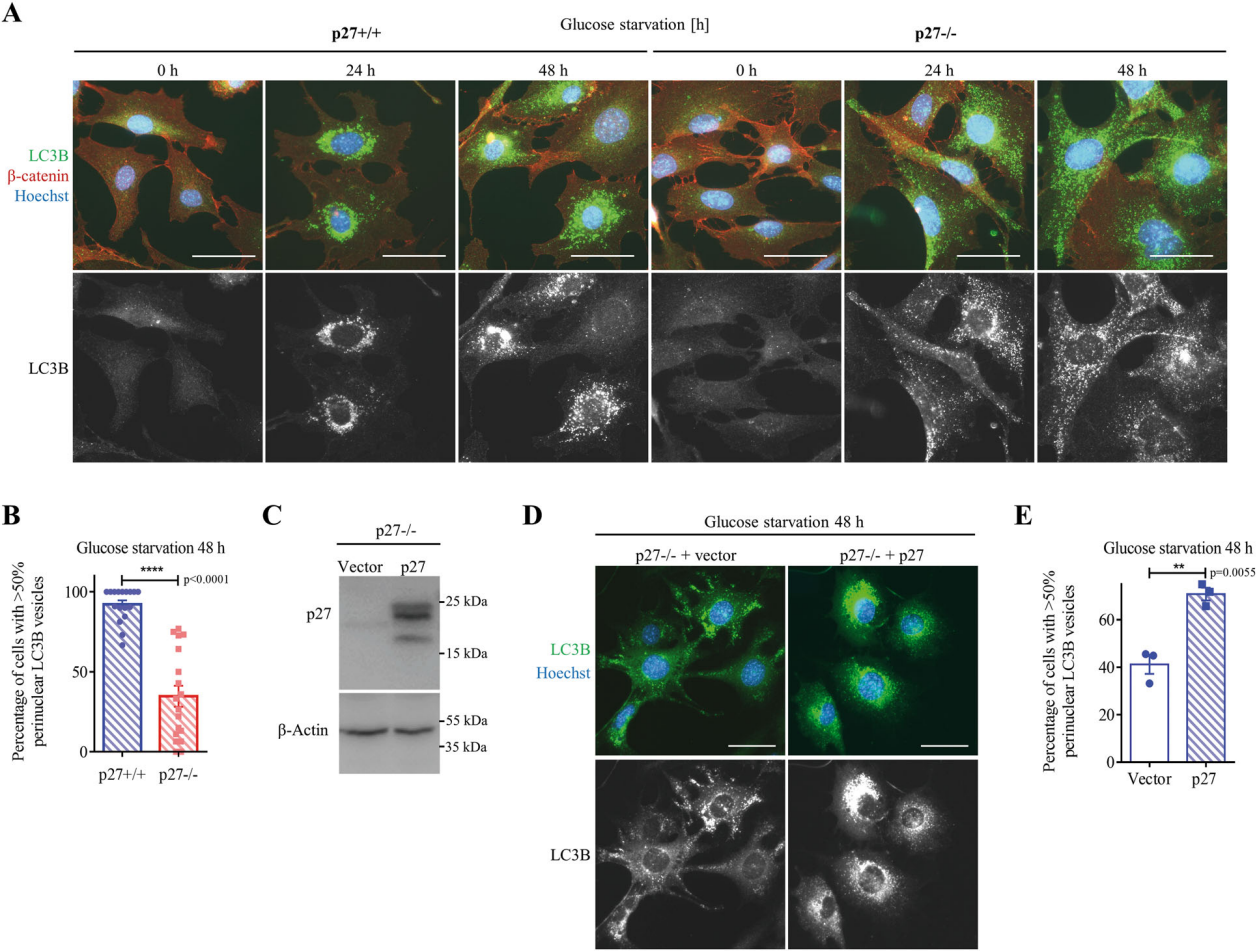
p27 affects autophagosome positioning in glucose-deprived cells. **A** p27^+/+^ and p27^−/−^ MEFs were glucose deprived for the indicated times and immunostained for LC3B. β-catenin was used to visualize cell shape and DNA was stained with Hoechst 33342. Scale bars are 50 µm. **B** Mean percentage of cells with over 50% of LC3B^+^ vesicles in the perinuclear region. *n* = 17 (p27^+/+^) and *n* = 18 (p27^−/−^) images from three experiments as described in (**A**) were used for quantification. Autophagosome distribution was evaluated based on a percentage of LC3 integrated density in perinuclear area (designed by region of interest [ROI]) versus LC3 integrated density in the whole cell. Graph show means ± SEM. **C** p27 immunoblot of p27^−/−^ MEFs retrovirally infected with p27 or empty vector. β-Actin was used as loading control. **D** LC3B immunostaining of MEFs infected with p27 or empty vector glucose deprived for 48 h. DNA was stained with Hoechst 33342. Scale bars are 50 µm. **E** Mean percentage of cells with over 50% LC3B^+^ vesicles in the perinuclear region from experiments as described in (**D**) from *n* = 3 independent experiments. Autophagosome distribution was evaluated as in (**B**). Graph show means ± SEM. **B**, **E** Statistical significance was evaluated by two-tailed Student’s *t* test with Welch’s correction.

The delivery of autophagosomes to the perinuclear area is crucial for their fusion with lysosomes and for cargo degradation^25,30,33^. Remarkably, the distribution of LC3B^+^ vesicles was different in p27^−/−^ cells compared to p27^+/+^ MEFs (Fig. 3A). While LC3B^+^ vesicles were predominantly juxtanuclear in over 90% of p27^+/+^ MEFs, it was the case in only about a third of p27^−/−^ cells (Fig. 3B). Instead, in p27-null MEFs, LC3B^+^ vesicles were distributed throughout the cytoplasm (Fig. 3A). To confirm the effect of p27 on LC3B^+^ vesicle positioning, p27 was re-expressed in p27^−/−^ MEFs (Fig. 3C) and the presence of p27 restored a predominantly perinuclear localization of LC3B^+^ vesicles in glucose-starved cells (Fig. 3D, E). Taken together, these data suggest that p27 regulates autophagosome localization during glucose deprivation and that defective autophagosome movement in p27^−/−^ cells impairs autophagosome–lysosome fusion.

We recently found that in response to amino acid deprivation, p27 promotes autophagy by interfering with the assembly of the Ragulator complex, thereby participating in mTOR inhibition^21^. The lysosome-associated multiprotein complex BORC mediates MT-dependent trafficking of autophagosomes and lysosomes during autophagy and autophagosome–lysosome fusion^45,46^. Importantly, upon nutrient depletion, Ragulator inhibits BORC and promotes the juxtanuclear positioning of vesicles, thus facilitating autophagosome–lysosome fusion^47,48^. We therefore tested whether p27-mediated inhibition of Ragulator on lysosomes may contribute to the regulation of vesicle positioning in glucose-deprived cells. First, inhibition of mTOR signaling was confirmed in glucose deprived p27^+/+^ and p27^−/−^ MEFs (Fig. S3A, B). In these conditions, p27^−/−^ cells did not maintain elevated mTOR activity as observed under amino acid starvation^21^. In fact, p27^+/+^ cells display increased p70 S6K1 phosphorylation upon glucose withdrawal compared to p27^−/−^ cells, which is associated with autophagy-related mTOR re-activation^49^, as indicated by the inhibition of mTORC1 activity following CQ treatment in p27^+/+^ cells (Fig. S3, A, B). Analysis of LC3B^+^ vesicle localization in glucose-starved p27^+/+^ and p27^−/−^ MEFs transfected with LAMTOR1 siRNA (Figure S3C) revealed that LAMTOR1 silencing increased the peripheral distribution of autophagosomes in p27^+/+^ cells, as previously reported^47,48^, but had no effect on autophagosome localization in p27^−/−^ cells (Fig. S3D, E). These results indicate that p27 regulates autophagosome positioning in a LAMTOR1-independent manner in glucose-deprived cells.

### Microtubule acetylation determines autophagosome positioning and autophagy flux

**Fig. 4:**
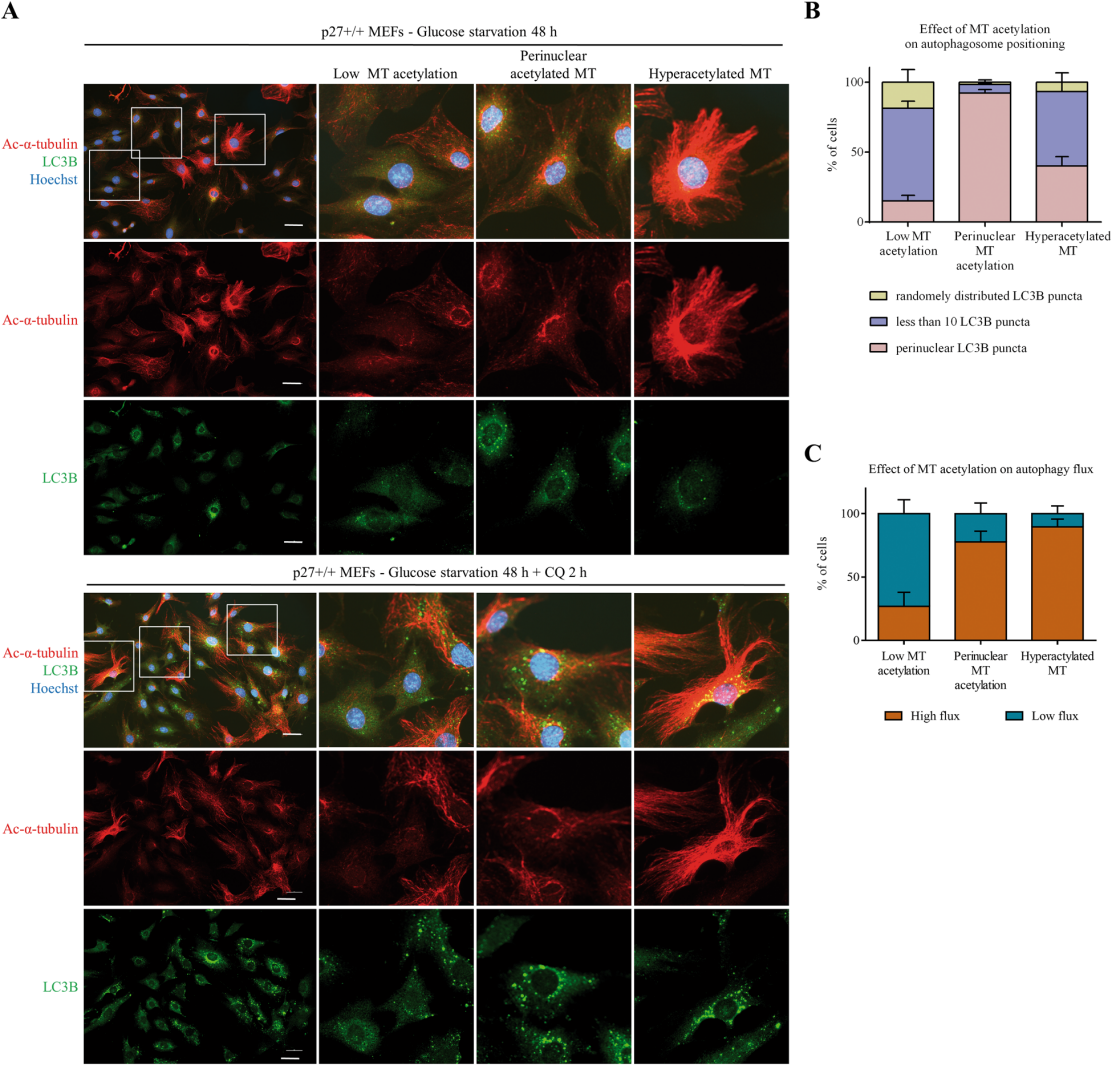
MT acetylation levels determine autophagosome positioning and autophagy flux. **A** Immunostaining for LC3B and acetylated (Ac-) α-tubulin of p27^+/+^ MEFs glucose starved for 48 h (upper panel) and treated with 50 µM CQ for the last 2 h (lower panel). Cells were classified into three categories in function of their microtubule (MT) acetylation pattern: low MT acetylation; perinuclear acetylated MT and hyperacetylated MT. In each population, LC3B^+^ vesicles were analyzed to determine if there is a correlation between MT acetylation status and vesicle positioning. Scale bars are 50 µm. **B** Mean percentage of cells with distinct LC3B distribution patterns (Randomly distributed, less than 10 LC3B puncta and perinuclear LC3B puncta) in each MT acetylation pattern described in (**A**). The graph shows means ± SEM from *n* = 2 independent experiments. At least 65 cells were analyzed per experiment. The number of LC3B puncta was determined using Find maxima function in ImageJ. **C** Autophagy flux (high or low) was determined based on the capacity of cells to accumulate LC3B dots upon CQ treatment in cells from experiments described in (**A**), in function of their MT acetylation status. Autophagy flux was estimated by determining the average LC3B fluorescence intensity in cells (designated as ROIs). Then, cells with a mean LC3B fluorescence intensity above that average were considered as having high autophagy flux and cells with a mean LC3B fluorescence intensity below that value were considered as having low autophagy flux. A total of 331 cells were analyzed from *n* = 2 independent experiments.

Autophagosome trafficking along MT tracks is mediated by motor proteins^27–29^. The recruitment of motor proteins to MT is controlled by MT stability and their post-translational modifications, such as acetylation at lysine 40 (K40)^31,32,50^. To test the importance of the MT network in glucose deprivation-induced autophagy, the correlation between MT acetylation levels, autophagosome positioning and autophagy flux was determined in p27^+/+^ MEFs (Fig. 4). Cells were immunostained for LC3B and acetylated-K40 α-tubulin (Ac-α-tubulin). Cells were classified into three categories in function of their MT acetylation status: low MT acetylation, perinuclear MT acetylation and hyperacetylated MT network (Fig. 4A). Then, the distribution and levels of LC3B^+^ vesicles were analyzed within each cell population (Fig. 4A, B). The same experiments were performed in the presence of 50 µM CQ to block autophagy and estimate the impact of MT acetylation on autophagy flux (Fig. 4A, C).

In cells with MT acetylation in the perinuclear area, LC3B^+^ vesicles were also perinuclear and CQ caused a strong accumulation of these vesicles, suggesting that these cells have high autophagy flux (Fig. 4). This is consistent with the idea that autophagosome–lysosome fusion occurs mostly in the proximity of the MTOC^30,45^. Cells with hyperacetylated tubulin had a low abundance of LC3B^+^ vesicles and their number dramatically increased upon CQ addition, indicative of a high autophagy flux (Fig. 4). Importantly, LC3B puncta appeared predominantly in the perinuclear zone following CQ treatment, reinforcing the idea that autophagosomes are delivered in the proximity of the centrosome for efficient fusion with lysosomes. In contrast, cells with low MT acetylation levels also exhibited low numbers of LC3B^+^ vesicles, which only slightly increased (mostly in the peripheral area) upon CQ addition, suggesting a low autophagy flux in these cells (Fig. 4). Taken together, these data suggest that MT acetylation favors autophagosome trafficking towards the perinuclear zone and autophagy flux in glucose-deprived cells. Thus, MT acetylation does not appear to be necessary for autophagosome–lysosome fusion but increases its efficiency, as previously described^51^.

### p27 controls autophagosome positioning by increasing microtubule acetylation

**Fig. 5:**
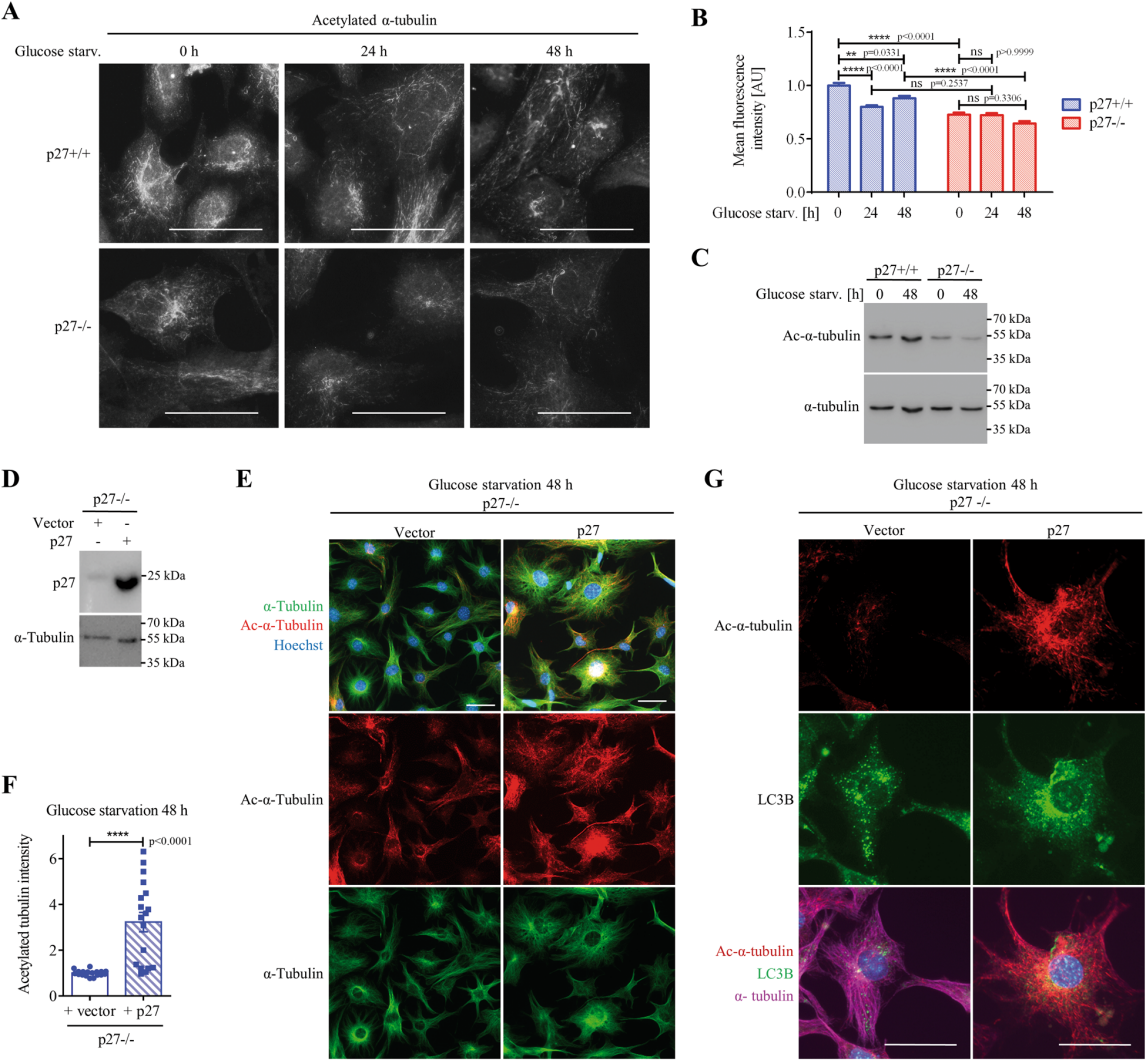
p27 promotes microtubule acetylation. **A** Acetylated (Ac-) α-tubulin immunostaining of p27^+/+^ and p27^−/−^ MEFs in full medium or glucose-deprived for 24 h and 48 h. **B** Quantification of acetylated tubulin intensity normalized to the mean value in p27^+/+^ cells in full medium from experiments described in (**A**). Number of cells used for quantification: p27^+/+^: *n* = 388 (0 h), *n* = 474 (24 h), *n* = 706 (48 h); p27^−/−^: *n* = 492 (0 h), *n* = 1316 (24 h), *n* = 689 (48 h). **C** Ac-α-tubulin and α-tubulin immunoblots in p27^+/+^ and p27^−/−^ MEFs in full medium or glucose deprived for 48 h. **D** p27 immunoblot in p27^−/−^ MEFs infected with either empty vector or p27. α-tubulin was used as loading control. **E** p27^−/−^ MEFs infected with either empty vector or p27 were glucose-starved for 48 h and immunostained for Ac-α-tubulin and α-tubulin. DNA was stained with Hoechst 33342. **F** Quantification of acetylated tubulin fluorescence intensity in p27^−/−^ MEFs re-expressing p27 normalized to control vector infected cells from *n* = 3 independent experiments as described in (**E**). At least 155 cells were analyzed per condition per experiment. **G** Ac-α-tubulin, LC3B and α-tubulin immunostaining of p27^−/−^ MEFs infected with either empty vector or p27, glucose starved for 48 h. **A**, **E**, **G** Scale bars are 50 µm. **B**, **F** Graphs show means ± SEM. Statistical significance was determined using 2-way ANOVA (**B**) or unpaired two-tailed Student’s *t* test with Welch’s correction (**F**).

Several reports have described the regulation of MT dynamics by p27, either through direct binding to MT or via the regulation of other proteins^11,50,52,53^. Although different mechanisms have been involved, the overall effect of p27 is to stabilize microtubules. Measurement of α-tubulin acetylation at K40 by immunofluorescence (Fig. 5A, B) and immunoblot (Fig. 5C) indicated that p27 promotes MT acetylation, as previously reported^11,50^, under both basal and glucose starvation conditions (24 h and 48 h). Furthermore, re-expression of p27 in p27^−/−^ cells was sufficient to increase MT acetylation (Fig. 5D–F) and to restore the perinuclear localization of autophagosomes in glucose starvation conditions compared to empty vector infected p27^−/−^ cells (Fig. 5G). Similar results were obtained in glucose-starved primary p27^+/+^ and p27^−/−^ MEFs in the presence of CQ, with p27^+/+^ cells exhibiting elevated MT acetylation levels and perinuclear autophagosome positioning, while p27^−/−^ MEFs had reduced MT acetylation levels and autophagosomes were distributed throughout the cytoplasm (Figure S4). Thus, p27 regulates MT acetylation and impaired LC3B^+^ vesicle trafficking in glucose starved MEFs appears to be a consequence of lower tubulin acetylation levels in these cells.

**Fig. 6:**
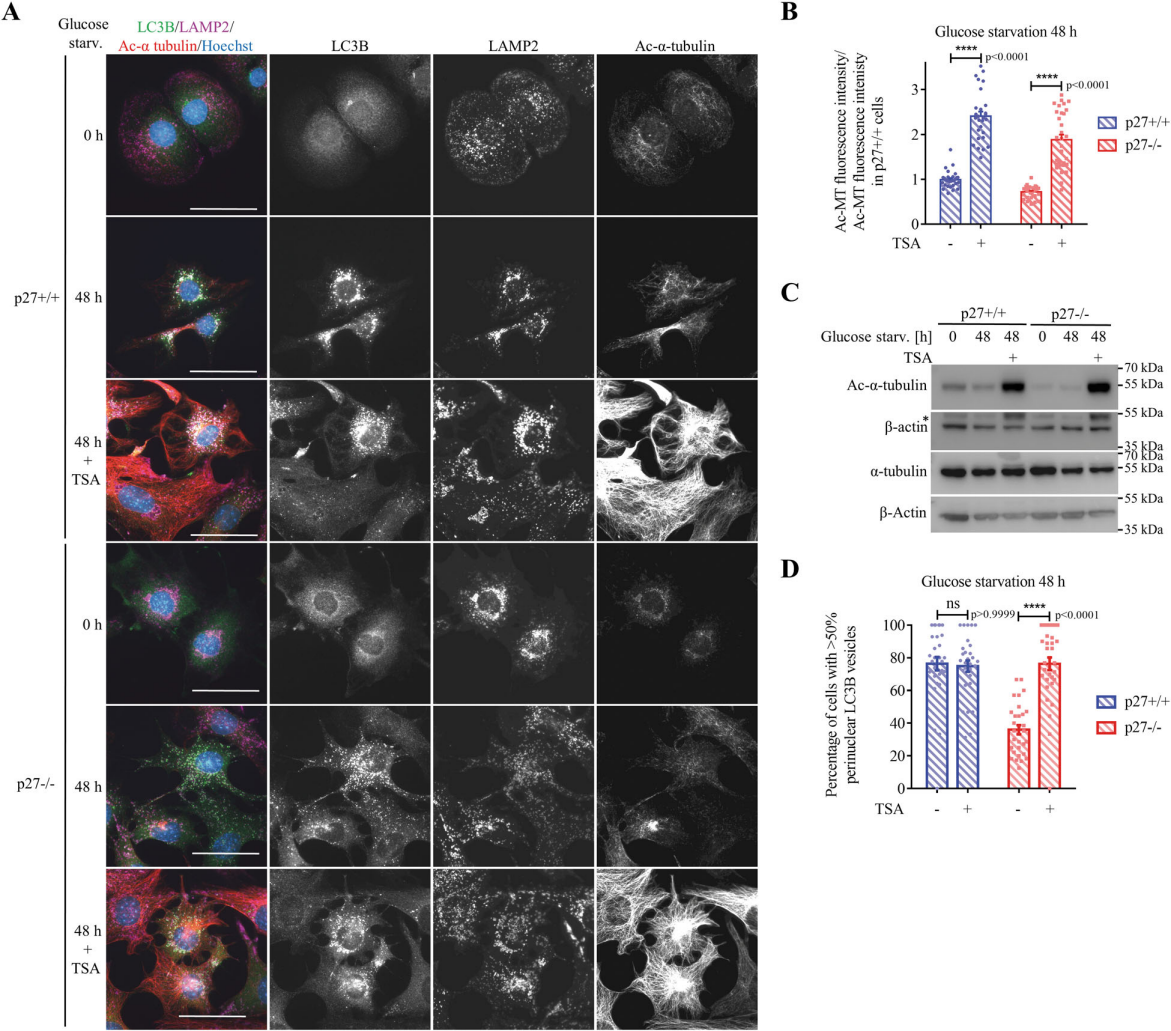
HDAC inhibition restores autophagosomes positioning in p27^−/−^ cells. **A** LC3B, LAMP2 and Ac-α-tubulin immunostaining of p27^+/+^ and p27^−/−^ MEFs glucose deprived for 48 h ± 0.2 µM TSA for 1 h. Scale bars are 50 µM. **B** Acetylated tubulin fluorescence intensity was measured in cells glucose-starved for 48 h. Values were normalized to 48 h glucose-starved p27^+/+^ cells. At least 160 cells were analyzed per condition in each experiment from *n* = 3 independent experiments. **C** Immunoblotting for Ac-α-tubulin and tubulin in cells as described in A. Actin was used as loading control. * denotes remaining Ac-α-tubulin signal after membrane stripping. **D** Percentage of p27^+/+^ and p27^−/−^ MEFs glucose deprived for 48 h ± 0.2 µM TSA for 1 h with >50 % of LC3B vesicles in the perinuclear region. Autophagosome distribution was evaluated based on a percentage of LC3 integrated density in perinuclear area (designed by region of interest [ROI]) versus LC3 integrated density in the whole cell. At least 91 cells were analyzed per condition. *n* = 3 independent experiments. **B**, **D** Bar graphs show mean ± SEM. Statistical significance was determined using 2-way ANOVA followed by Bonferroni multiple comparison test.

To determine the importance of MT acetylation in p27-mediated regulation of glucose starvation-induced autophagy, p27^+/+^ and p27^−/−^ MEFs were treated with the histone deacetylase (HDAC) inhibitor Trichostatin A (TSA). TSA was shown to prevent α-tubulin deacetylation by blocking HDAC6 activity^54^. TSA treatment dramatically increased MT acetylation (Fig. 6A–C), as expected, and induced the accumulation of LC3B^+^ vesicle around the nucleus in p27^−/−^ cells (Fig. 6A, D). In addition, while TSA treatment did not affect autophagy rate in full medium (Fig. S5A, B, D, G), suggesting that microtubule acetylation is dispensable for basal autophagy, TSA rescued impaired autophagosome maturation and autophagy flux in p27^−/−^ cells in glucose-deprivation conditions (Fig. S5A, C, E, H), without significant effect in p27^+/+^ MEFs in which tubulin acetylation levels are already elevated. On the other hand, TSA did not rescue the autophagy defect of p27^−/−^ MEFs upon amino acid starvation (Fig. S5F, I), although it increased autophagy flux in p27^+/+^ cells, albeit not significantly. Consistent with the positive effect of TSA on autophagosome maturation, we observed a marked reduction in p62 levels in p27^−/−^ MEFs, indicative of a restored autophagy flux (Fig. S6). Taken together, these data suggest that p27 regulates autophagy flux via a MT acetylation-dependent mechanism in glucose-deprived cells.

### p27 increases microtubule acetylation via stabilization of ATAT1

**Fig. 7:**
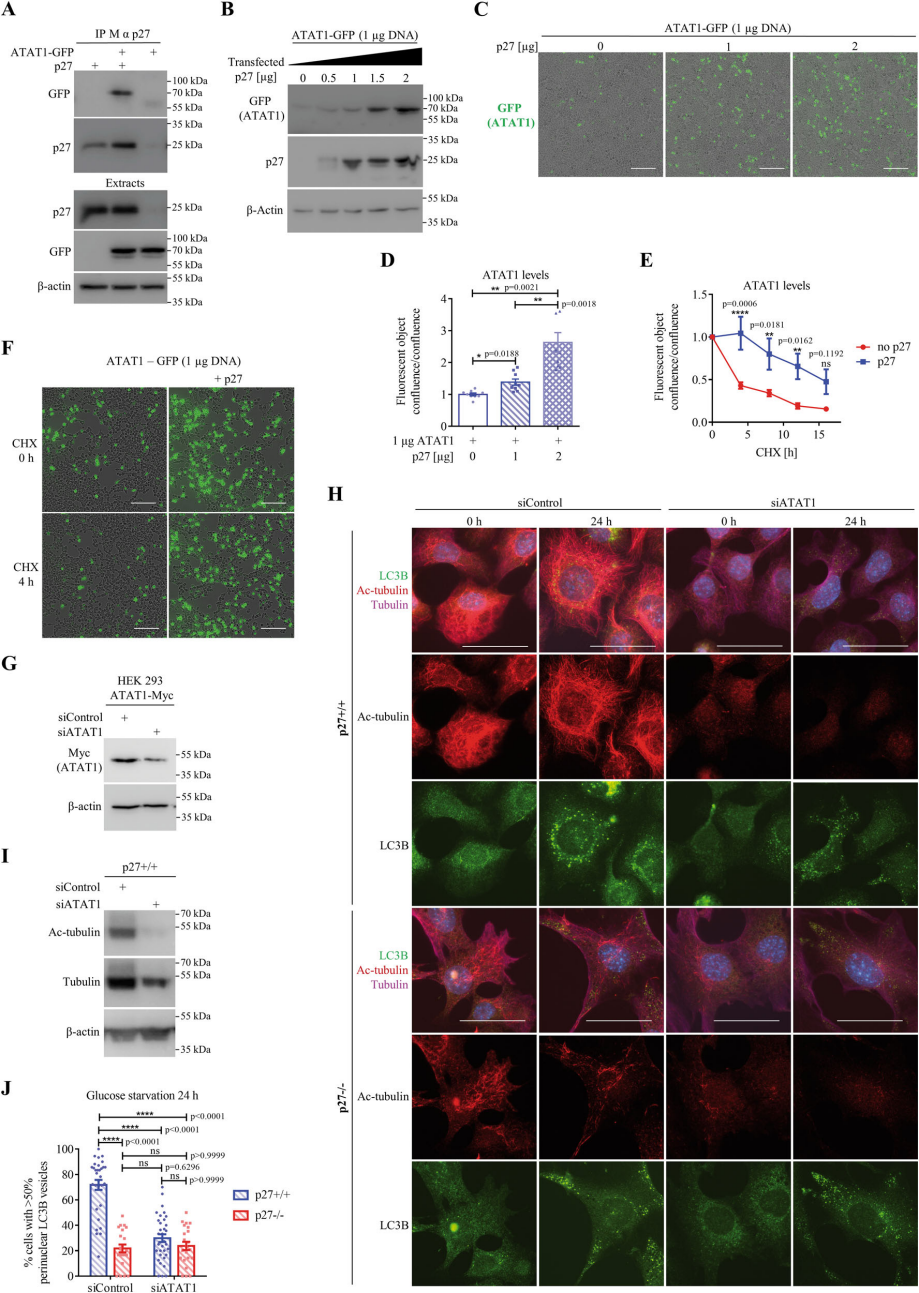
p27 regulates autophagosome positioning via the control of ATAT1 stability. **A** HEK 293 cells were transfected with p27 and/or ATAT1-GFP for 24 h. p27 was immunoprecipitated and p27-bound ATAT1 was detected with anti-GFP antibodies. Expression levels of p27 and ATAT1-GFP were determined by immunoblotting in extracts. β-actin was used as loading control. **B** HEK 293 cells were transfected with 1 µg of ATAT1-GFP vector and increasing amounts of p27 for 24 h. Expression levels of ATAT1-GFP and p27 were determined by immunoblotting. β-actin was used as loading control. **C** Representative images of cells from (**B**) acquired with the IncuCyte. Green fluorescence represents ATAT1 expression. Scale bars are 200 µm. **D** Quantification of experiments as described in (**C**). Fluorescent object confluence was measured and normalized to cell confluence (phase contrast). Values were normalized to empty vector transfected cells condition. *n* = 9 images per condition from three independent experiments. **E** HEK 293 cells were transfected with 1 µg ATAT1-GFP for 48 h and 2 µg p27 or empty vector and treated with 50 µg/ml cycloheximide (CHX) for 16 h. Images were acquired with an IncuCyte every 4 h to monitor ATAT1-GFP expression levels (green fluorescence). Values of fluorescent object confluence were normalized to cell confluence. *n* = 12 images per time point for each condition from three independent experiments were used for quantification. **F** Representative images of experiment as described in (**E**). Scale bars are 200 µm. **G** Validation of ATAT1 siRNA. HEK 293 cells were transfected with Myc-ATAT1 for 24 h and then with 50 nM siRNA for another 48 h and subjected to immunoblot against Myc. Actin was used as loading control. **H** p27^+/+^ and p27^−/−^ MEFs were transfected with either control or ATAT1 siRNA. After 24 h, cells were collected and fixed (0 h) or glucose starved for 24 h. Cells were stained for LC3B, acetylated α-tubulin and α-tubulin. Scale bars are 50 µM. **I** Acetylated (Ac-) α-tubulin and α-tubulin immunoblot on p27^+/+^ MEFs transfected with ATAT1 siRNA and collected after 48 h. Since tubulin levels change after ATAT1 siRNA transfection, β-actin was used as loading control. **J** Mean percentage of cells with over 50% of perinuclear LC3B^+^ vesicles from three (p27^+/+^) or two (p27^−/−^) experiments as described in (**H**). Autophagosome distribution was evaluated based on a percentage of LC3B integrated density in perinuclear area (designated as region of interest [ROI]) versus LC3B integrated density in the whole cell. Number of images used for quantification: p27^+/+^: *n* = 31 (siControl), *n* = 34 (siATAT1); p27^−/−^: *n* = 22 (siControl), *n* = 22 (siATAT1). At least 105 cells per condition were analyzed per experiment. **D**, **E**, **J** Graphs show means ± SEM. Statistical significance was determined by 1-way (**D**) or two-way ANOVA (**E**, **J**) followed by Bonferroni multiple comparison test.

**Fig. 8:**
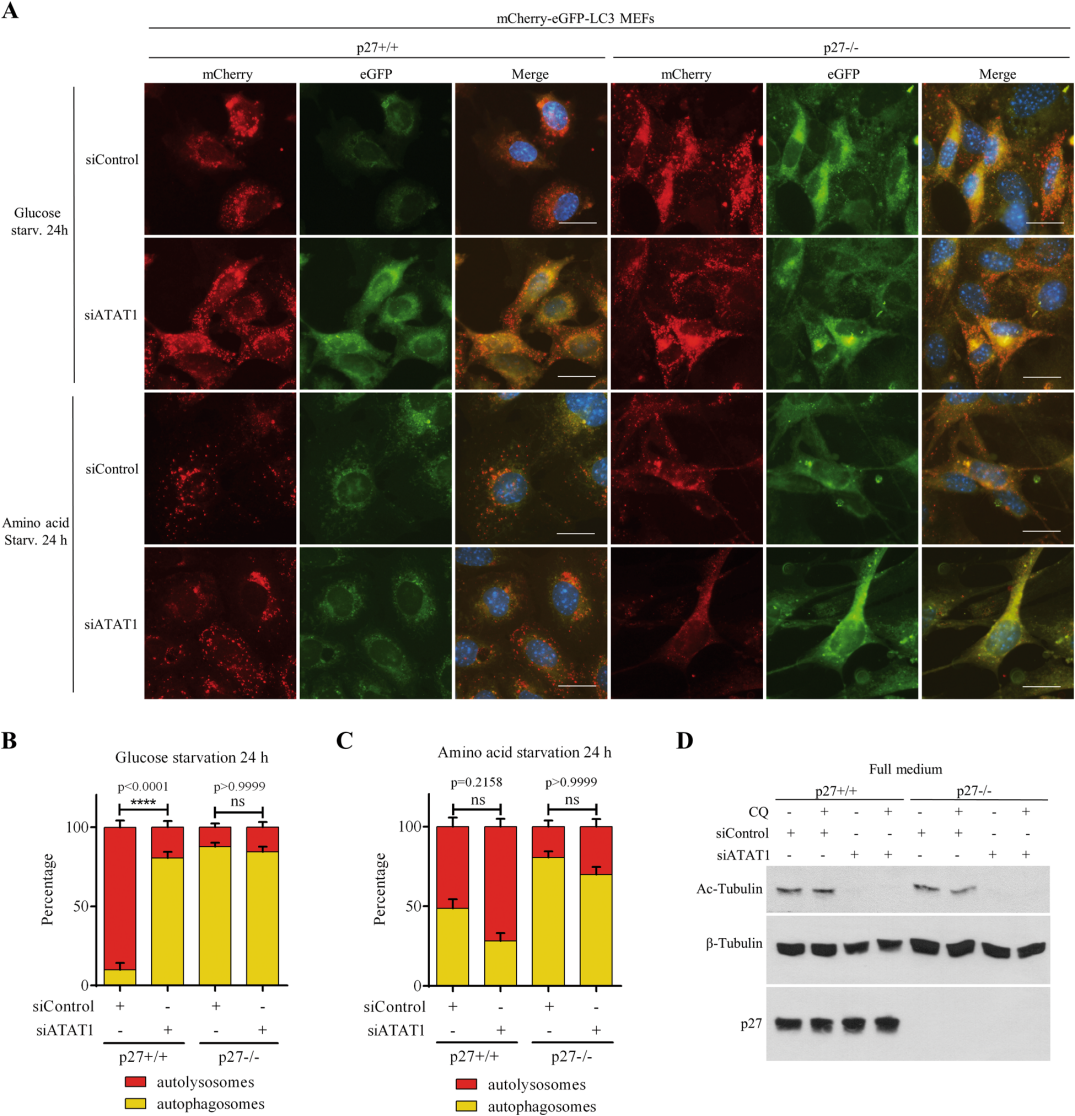
ATAT1 mediates p27 regulation of autophagy in glucose-deprived cells. **A** p27^+/+^ and p27^−/−^ mCherry-eGFP LC3 MEFs were transfected with siRNA against ATAT1 or control siRNA. After 24 h, cells were starved for either glucose or aa for further 24 h. Scale bar 50 µm. **B**, **C** Graphs show the percentage of autophagosomes (yellow puncta) and autolysosomes (red puncta) in glucose-deprived cells (**B**) and amino acid-deprived cells (**C**), as described in (**A**). Number of analyzed images: Glucose starvation: p27^+/+^ siControl *n* = 31; p27^+/+^ siATAT1 *n* = 45; p27^−/−^ siControl *n* = 38, p27^−/−^ siATAT1 *n* = 49; Amino acid starvation: p27^+/+^ siControl *n* = 40; p27^+/+^ siATAT1 *n* = 34; p27^−/−^ siControl *n* = 38, p27^−/−^ siATAT1 *n* = 57 Statistical significance was determined by 2-way ANOVA followed by Bonferroni multiple comparison test. **D** Immunoblot showing the efficiency of siATAT1 by monitoring microtubule acetylation levels in p27^+/+^ and p27^−/−^ MEFs. Membranes were probed for Ac-α-tubulin, p27 and β-tubulin as loading control.

p27 was recently reported to interact with the MT acetylating enzyme α-tubulin acetyltranferase (ATAT1) and to increase its stability, which was important in regulating axonal trafficking in cortical and motor neurons^50^. We confirmed the physical interaction between p27 and ATAT1 by co-immunoprecipitation (co-IP) experiments (Fig. 7A). Co-transfection of increasing amounts of p27 increased ATAT1 protein levels (Fig. 7B–D), consistent with p27 stabilizing ATAT1^50^. This was confirmed by cycloheximide treatment to block protein translation and determine the half-life of ATAT1 by measuring ATAT-GFP fluorescence with an IncuCyte in the presence or absence of p27 expression in HEK 293 cells (Fig. 7E, F). While ATAT1 had a half-life of about 4 h in the absence of p27, it increased to approximately 16 h upon p27 overexpression, as previously reported^50^. Since ATAT1 regulates MT-dependent axonal transport during neurogenesis^50^, we tested if the same mechanism was implicated in the regulation of autophagosome trafficking in glucose starved MEFs. Autophagosome positioning was analyzed in glucose-deprived p27^+/+^ and p27^−/−^ MEFs transfected with ATAT1 siRNA. Since we do not have a functional antibody against endogenous ATAT1, siRNA efficiency was verified against a myc-tagged ATAT1 (Fig. 7G). ATAT1 knockdown dramatically reduced MT acetylation (Fig. 7H, I), as expected, and significantly decreased the localization of LC3B^+^ vesicles in the perinuclear area in p27^+/+^ MEFs, whereas it did not affect the already randomly distributed LC3B^+^ vesicles in p27^−/−^ cells (Fig. 7H, J). The depletion of ATAT1 in MEFs, validated by monitoring MT acetylation levels (Fig. 8D), resulted in reduced autolysosome formation in p27^+/+^ cells without affecting p27^−/−^ cells (Fig. 8A, B), in which autophagosome maturation is already impaired. ATAT1 knockdown did not significantly affect autophagosome maturation in amino acid-deprived cells (Fig. 8A, C), consistent with the idea that the regulation of autophagy in response to glucose or amino acid starvation by p27 is mediated by distinct mechanisms (Figure S3 and^21^). Collectively, these results indicate that in glucose-deprived cells, p27 promotes autophagy by controlling autophagosome positioning via ATAT1-dependent MT acetylation.

## Discussion

Several lines of evidence indicate that cellular energy status controls p27 levels and localization^17,20,37,44,55–57^. In response to glucose starvation, a fraction of p27 relocalizes to the cytoplasm and this is accompanied by a mild increase in p27 levels^17,20,37,44,56^. However, some reports observed p27 degradation following its cytoplasmic relocalization in glucose-deprived cells^55,57^. These differences may reflect the use of distinct cellular models and that starvation was performed in low glucose conditions^55,57^, instead of glucose-free medium and dialyzed serum as in the present study. The cytoplasmic accumulation of p27 in glucose-deprived cells may seem surprising. Given its role as a cyclin/CDK inhibitor in the nucleus, one would expect the maintenance of p27 nuclear localization in conditions of metabolic stress to enforce cell cycle arrest, as observed in serum-deprived cells^58^. Nevertheless, the remaining nuclear p27 likely still contributes to G1 cell cycle arrest upon glucose deprivation^17,56^. Thus, both cytoplasmic and nuclear p27 appear to cooperate to adapt the cellular response to extracellular conditions by causing cell cycle arrest and promoting autophagy to cope with metabolic stress. Indeed, cells lacking p27 cannot face this challenge and undergo apoptosis^17,18,21^. Interestingly, lysosomal activity and autophagy were recently found to control the depth of the quiescence state^59^ and an attractive hypothesis is that p27 could contribute to establishing quiescence by inhibiting cyclin-CDKs and maintaining cells in a shallow, easily reversible quiescence state by promoting autophagy.

Glucose withdrawal was shown to induce autophagy in a p27-dependent manner^17^. Our results confirm this pro-autophagic function and indicate a role for p27 in autophagic vesicle positioning. In wild-type cells, autophagosomes are located mostly in the perinuclear zone of the cell, whereas in p27^−/−^ cells, LC3B^+^ vacuoles were distributed throughout the cytoplasm. During autophagy, autophagosomes form randomly in the cell periphery and then move along MT towards the centrosome where they fuse with lysosomes^25,30,33,34^. Thus, the distribution of autophagosomes in the cell is crucial for their maturation and autophagy flux. p27 was shown to stabilize MT by several mechanisms and promote their acetylation by stabilizing ATAT1^11,50,52^. p27 promotes MT stability either through direct binding to MT^52^ or by its interaction with MAPs, such as Stathmin^11,57^, a MT destabilizing protein whose activity is inhibited by p27, or PRC1^53^, a MAP that bundles antiparallel MT. Since the effect of p27 on MT requires its cytoplasmic localization, one would expect MT acetylation to increase in glucose-deprived cells, in which there are more p27 in the cytoplasm. MT acetylation in basal and starvation conditions is more abundant in p27^+/+^ than in p27^−/−^ MEFs, but it tends to decrease during starvation, unlike what was reported previously^60^. These differences are probably due to differences in cellular models or starvation protocol. Geeraert et al. used EBSS starvation (low glucose, no amino acids, no serum) for 2 h^60^, whereas in this study, MEFs were glucose starved in the presence of dialyzed serum and amino acids for 48 h.

Although MT acetylation on K40 is considered as a hallmark of MT stability, MT acetylation is a consequence and not a cause of MT stability^61^. MT acetylation was shown to facilitate autophagy^25,34,36,60^. To determine if p27-mediated autophagy was dependent on MT acetylation, MT stability, or both, we used the HDAC inhibitor TSA that increases MT acetylation without affecting their stability^61^. Treatment of glucose-deprived p27^−/−^ MEFs with TSA rescued their autophagosome positioning defect and restored autophagy flux, indicating that MT acetylation, and not their stability, is required for retrograde transport of autophagosomes, consistent with previous studies^36,62–64^. Intriguingly, genetic depletion of HDAC6 inhibits autophagosome maturation and fusion under basal conditions but not upon starvation^62,65^. However, mice lacking HDAC6 are viable and develop normally^66^, whereas autophagy-deficient mice die perinatally^67^, indicating that HDAC6 is dispensable for autophagy. Nevertheless, given the multiple functions of HDACs in the cell, results obtained with pharmacological HDAC inhibitors should be interpreted with caution. Indeed, HDAC inhibition not only affects MT acetylation but also that of many genes and proteins, including ULK1, ATG5, ATG7, and LC3B, which may either promote or inhibit autophagy^68–70^. Although most of these proteins are targeted by sirtuin deacetylases (class III HDACs), which are not affected by TSA^69,71^, ATG7 is acetylated by HDACs sensitive to TSA^72^. Importantly, nuclear HDACs may epigenetically regulate the expression of autophagy-related genes and deacetylation of histone H3 and H4 promotes autophagy^73,74^. Thus, TSA may affect the autophagy machinery in different ways, in addition to its effect on MT. To specifically investigate the importance of MT acetylation on autophagy, we used siRNA against ATAT1, the enzyme responsible for K40 acetylation of α-tubulin^75,76^. MT acetylation increases the recruitment of motor proteins to MT tracks, thereby affecting intracellular trafficking^31,32,36^. p27 binds to and stabilizes ATAT1^50^ and ATAT1 silencing disrupts the perinuclear localization of autophagosomes in cells expressing p27 and impairs their autophagy flux, suggesting that p27 regulates autophagy via ATAT1.

Despite many studies investigating starvation-induced autophagy, few of them have addressed the fact that autophagy mechanisms differ in function of the type of metabolic stress. Investigation of the mechanism of amino acid deprivation-induced autophagy revealed the regulation of mTORC1 signaling and the Ragulator complex by p27^21^. In contrast, our results indicate that in glucose-deprived cells, p27 promotes autophagy in a Ragulator-independent manner. Furthermore, neither TSA treatment nor ATAT1 silencing significantly affects amino acid starvation-induced autophagy, confirming that distinct molecular mechanisms are at play in the regulation of autophagy in response to amino acid or glucose starvation. This is consistent with previous studies showing that signaling pathways leading to autophagosome formation are different in response to amino acid and glucose withdrawal. While the former induces autophagy via the canonical pathway mediated by PI(3)P and ULK1/2, the latter generates PI(5)P to induce ATG recruitment to pre-autophagosomal sites in an ULK-independent manner^77,78^. Further studies have shown that both types of starvation trigger TFEB nuclear localization and autophagy induction, but TFEB is regulated by different upstream kinases. In amino acid-deprived cells, mTORC1 inhibition prevents the direct phosphorylation of TFEB that maintains its cytoplasmic localization^79^. In contrast, in glucose-deprived cells, mTORC2 activates AKT, which in turn inhibits GSK3β activity, preventing TFEB phosphorylation^80^. Finally, our results indicate that basal autophagy is not affected by p27 status or MT acetylation levels. Previous studies have shown differences between basal and stress-induced autophagy, notably the site of autophagosome formation^81^, upstream signaling governing autophagy initiation^82^, and factors controlling autophagosome maturation^65^. Altogether, it appears that distinct metabolic pathways are interconnected with autophagy to adapt autophagy rate to environmental nutrient availability.

The regulation of autophagic vesicle trafficking by cytoplasmic p27 may have therapeutic relevance. In neuronal cells, axonal transport of autophagosomes is important for cargo degradation and autophagy defects lead to neurodegenerative diseases^36^. In line with this, p27 has been involved in neural development^50,52,83^. In cancer, autophagy may either prevent or promote tumorigenesis^22,23^. Similarly, p27 may act as a tumor suppressor or oncogene and its cytoplasmic localization appears to promote cancer development and progression and is associated with poor prognosis^7,8,10^. Since p27-mediated autophagy confers resistance to metabolic stress caused by glucose starvation^17^, it could be one of the mechanisms by which p27 contributes to tumor progression. Therefore, targeting oncogenic kinases causing p27 cytoplasmic localization, preventing microtubule acetylation, or inhibiting autophagy could overcome chemoresistance of tumors with cytoplasmic p27.

## Materials and Methods

### Antibodies, reagents, and plasmids

Mouse anti p27 (F8, sc-1641), p27 (SX53G8.5, sc-53871), and rabbit anti p27 (C19, sc-528), Myc (A14, sc-789), p62/SQSTM1 (H290, sc-25575) antibodies were purchased from Santa Cruz Biotechnology. Mouse anti p27 (610242) and Grb2 (610112) antibodies were purchased from BD-Transduction Laboratories. Mouse anti β-actin (A2228), and β-tubulin (T4026), α-tubulin-FITC (F2168), Acetyl-K40 α-tubulin (T6793) antibodies were purchased from Sigma-Aldrich. Rabbit anti LC3A/B (#4108), AMPK (#8532), p-AMPK (T172) (#2535), phospho-p70 S6K1 (Thr389) (#9234), p70 S6K1 (#27087) and LAMTOR1 (#8975) antibodies were purchased from Cell Signalling Technology. Rabbit anti ATG5 (GTX113309**)** antibodies were purchased from Genetex. Rabbit anti phospho-Ser278 ATG16L1 (ab195242) antibodies were purchased from Abcam. Mouse anti LC3B (0231-100/LC3-5F10) was purchased from NanoTools. Secondary antibodies against Ig conjugated to horseradish peroxydase or Cyanine-2 and -3 were purchased from Jackson ImmunoResearch. siRNA control (sc-108727), siRNA against mouse LAMTOR1 (sc-37007) and ATAT1 (sc-108799) were purchased from Santa Cruz Biotechnology. Chloroquine diphosphate (C6628) and Trichostatin A (T1952) were purchased from Sigma-Aldrich.

p27 constructs and p27 point mutants and deletion mutants in pCS2 + , pcDNA3.1+Hygro (Invitrogen), pQCXIP (Clontech), pBabe puro, pWZL-Blast were described previously^9,84^. pEF5B-FRT-GFP-ATAT1 was a gift from Maxence Nachury (Addgene #27099)^76^. GFP-ATAT1 was subcloned into pQCXIP. pBabe-puro-mCherry-eGFP-LC3B was a gift from Jayanta Debnath (Addgene #22418)^85^. All plasmids were verified by DNA sequencing.

### Cell culture and transfections

All cells were incubated at 37 °C and 5% CO_2_ in DMEM (D6429, Sigma), 4.5 g/l glucose supplemented with 10% fetal bovine serum [FBS], 0.1 mM nonessential amino acids and 2 µg/ml penicillin–streptomycin. Primary MEFs were isolated from p27^+/+^ or p27^−/−^ embryos and immortalized by retroviral infection with a vector encoding the human papilloma virus E6 protein and hygromycin resistance, as previously^9,12^. Two days after infection, cells were selected with appropriate antibiotics at following concentrations: 2 µg/mL puromycin, 250 µg/mL hygromycin, 16 µg/mL of blasticidin. Cells were kept under selection at all times. HEK 293 cells were authenticated by short tandem repeat profiling. All cells were mycoplasma free as estimated by routine DAPI staining. For glucose deprivation, cells were rinsed twice with PBS and once with glucose-free DMEM (D5030, Sigma-Aldrich) supplemented with 0.1 mM nonessential amino acids, 2 µg/ml penicillin–streptomycin, 2mM L-glutamine and 10% dialyzed FBS. FBS was dialyzed at 4 °C in 3,500 MW cut-off dialysis tubing (SpectrumLabs, 132111) against PBS for 6 h and overnight. Pharmacological inhibitors were used at the following concentrations: Chloroquine 50 µM for 2 h (immunofluorescence) or 24 h (immunoblotting) in LC3B turnover assays as indicated in figure legend; TSA 200 nM for 1 h. Control cells were treated with the same volume of corresponding vehicle. For co-immunoprecipitations, HEK 293 cells were transfected by the calcium phosphate method and collected 24 h post-transfection. siRNAs were transfected for 48 h before starvation using Interferin (Polyplus transfection) according to manufacturer’s instructions.

### Immunofluorescence

MEFs were seeded on coverslips and grown to 50-60% confluence. Cells were rinsed and starved for 24–48 h prior to fixation with 1% PFA for 3 min at room temperature followed by 100% methanol for 5 min at −20 °C. Coverslips were washed three times 5 min with PBS and blocked for 20 min with blocking solution (PBS, 3% BSA, 0.05% Tween 20 and 0.08% sodium azide) at room temperature. Then, coverslips were incubated with primary antibodies diluted in blocking solution for 1 h at 37 °C, washed three times 5 min in PBS, and incubated with Cy2, Cy3 or Cy5-conjugated secondary antibodies for 30 min at 37 °C. Next, coverslips were washed three times 5 min in PBS, with the first wash containing 0.1 μg/mL Hoechst H33342. Where indicated, coverslips were incubated with α-tubulin-FITC antibody prior to H33342 staining. Coverslips were mounted on glass slides using gelvatol (10% polyvinyl alcohol (w/v), 20% glycerol (v/v), 70 mM Tris pH 8). Image acquisition was performed on a Nikon 90i Eclipse microscope with DS-Qi2 HQ camera and the NIS Element BR software. NIS, ZEN 3.1 Blue and ImageJ 1.51 W were used for quantifications. To measure fluorescent intensity of specific cellular compartments, regions of interest (ROI) were delineated and the signal was analyzed within the ROI. To determine the positioning of autophagosome, LC3B integrated density within perinuclear ROI was expressed as percentage of whole cell LC3B integrated density. Cells with more than 50% of LC3B signal in perinuclear ROI were counted as cells with predominantly perinuclear autophagosomes. To estimate autophagy flux by IF, the average LC3B fluorescence intensity was determined in cells treated with CQ. Cells with mean fluorescence intensity higher than this average value were considered having high autophagy flux, while cells with mean fluorescence intensity lower than this value were considered as having low autophagy flux. Similarly, acetylated tubulin fluorescence intensity was evaluated by comparing to a threshold corresponding to average microtubule acetylation in p27^+/+^ cells. Cells with MT acetylation below or above this value were considered as cells with low microtubule acetylation or hyperacetylation, respectively. Cells in which MT hyperacetylation was observed only in the perinuclear zone were considered as cells with perinuclear MT acetylation. The numbers of LC3B, p-ATG16L1 and ATG5 puncta were determined using Find maxima function in ImageJ.

### Immunoprecipitation

Cells were lysed in IP buffer (150 mM NaCl, 50 mM HEPES pH 7.5, 1% NP-40, 1 mM EDTA, 2.5 mM EGTA, 0.1% Tween20 and 10% glycerol, complemented with 1 mM DTT, 10 mM β-glycerophosphate, 10 mM NaF, 10 mM sodium orthovanadate, and 10 µg/ml Aprotinin, 10 µg/ml Bestatin, 10 µg/ml Leupeptin and 10 µg/ml Pepstatin A). Lysates were sonicated for 10 s, centrifuged for 5 min at 12,500 rpm and supernatants were collected. Protein concentrations were determined by Bradford assay and 500 µg of proteins were incubated with 12 µl protein-A sepharose beads (IPA300, Repligen) and 3 µg of indicated antibodies. Antibody-beads complexes were rinsed 4 times in IP buffer and eluted in 10 µl 4X Laemmli buffer (8%SDS, 40% Glycerol, 240 mM Tris-HCl pH 6.8, 400 mM DTT). Samples were boiled 3 min at 96 °C prior to loading onto SDS-PAGE gel. Immunoblotting of 1/10^th^ of input was used to determine amounts of respective proteins in lysates and for loading control.

### Immunoblot

Cells were lysed in 2X Laemmli buffer, sonicated twice for 15 s and boiled 3 min at 96 °C. Proteins were quantified by micro BCA assay (Thermo Scientific Pierce, 23235) and 20–30 µg of proteins were loaded on SDS-PAGE gel following the addition of DTT at 200 mM final concentration. After electrophoresis, proteins were transferred to polyvinylidene difluoride membrane (Immobilon-P, Millipore). Membranes were blocked with PBS-T (PBS, 0.1% Tween-20), 5% non-fat dry milk prior to incubation with primary antibodies overnight at 4 °C. The following day, membranes were rinsed thrice with PBS-T and incubated with HRP-conjugated secondary antibody for 4–6 h at room temperature. Signal was visualized with enhanced chemiluminescence detection reagents (Millipore, BioRad, and Ozyme) on autoradiographic films (Blue Devil) or with a Fusion Solo S (Vilber) digital acquisition system. Densitometry analyses were performed with the ImageJ software. Proteins of interests were normalized to loading control (β-Actin, β-Tubulin, or Grb2) and phospho-protein signals were normalized to total levels of the corresponding protein. To determine autophagy flux, the ratio of LC3B-II/loading control in the presence of CQ by that in the absence of CQ was calculated.

### Autophagy turboGFP-LC3B IncuCyte assay

Cells expressing turboGFP-LC3B were seeded (5000 cells/well in 96-well plates) and grown overnight in MEM (Sigma, 56419 C) medium supplemented with 4.5 g/l glucose, 10% FBS, 0.1 mM nonessential amino acids and 2 µg/ml penicillin–streptomycin before starvation as described above. Images were acquired with an Incucyte FLR system equipped with a 20x objective and tGFP intensity was measured as a ratio between fluorescence object confluence (green fluorescence) and confluence (phase contrast) using the Incucyte software. Values were normalized to fluorescence values in the first scan (0 h), which was acquired prior to starvation.

### Determination of ATAT1 half-life

HEK 293 cells were transfected with ATAT1-GFP and/or p27 encoding plasmids for 48 h and treated with 50 µg/ml cycloheximide (CHX) for 16 h. GFP fluorescence intensity was monitored using the IncuCyte at intervals of 4 h. The fluorescence signal was normalized to cell confluence at each time point using the Incucyte software.

### Statistical analyses

Data were analyzed using the Graphpad Prism 6.0 software. Statistical significance between three groups or more were determined using ANOVA test followed by Bonferroni test for multiple comparisons. Difference between two groups was evaluated using unpaired two-tailed *t* test with Welch’s correction. Data are presented as mean ± SEM. Symbols used are ns: *p* > 0.05; *: *p* ≤ 0.05; **: *p* ≤ 0.01; ***: *p* ≤ 0.001; ****: *p* ≤ 0.0001.

## Supplementary information

Figure S1

Figure S2

Figure S3

Figure S4

Figure S5

Figure S6

Dataset Fig. 1

Dataset Fig. 2

Dataset Fig. 3

Dataset Fig. 4

Dataset Fig. 5

Dataset Fig. 6

Dataset Fig. 7

Dataset Fig. 8

Dataset Fig. S1

Dataset Fig. S2

Dataset Fig. S3

Dataset Fig. S4

Dataset Fig. S5

Dataset Fig. S6

## Data Availability

The datasets used and/or analyzed during the current study are included with this published article as supplementary information files or are available from the corresponding author on reasonable request.
