# Supplementary figures and images for: p27 controls autophagic vesicle trafficking in glucose-deprived cells via the regulation of ATAT1-mediated microtubule acetylation
Source: Cell Death Dis. 2021 May 13;12(5):481. doi: 10.1038/s41419-021-03759-9 (PMC8119952; doi:10.1038/s41419-021-03759-9)

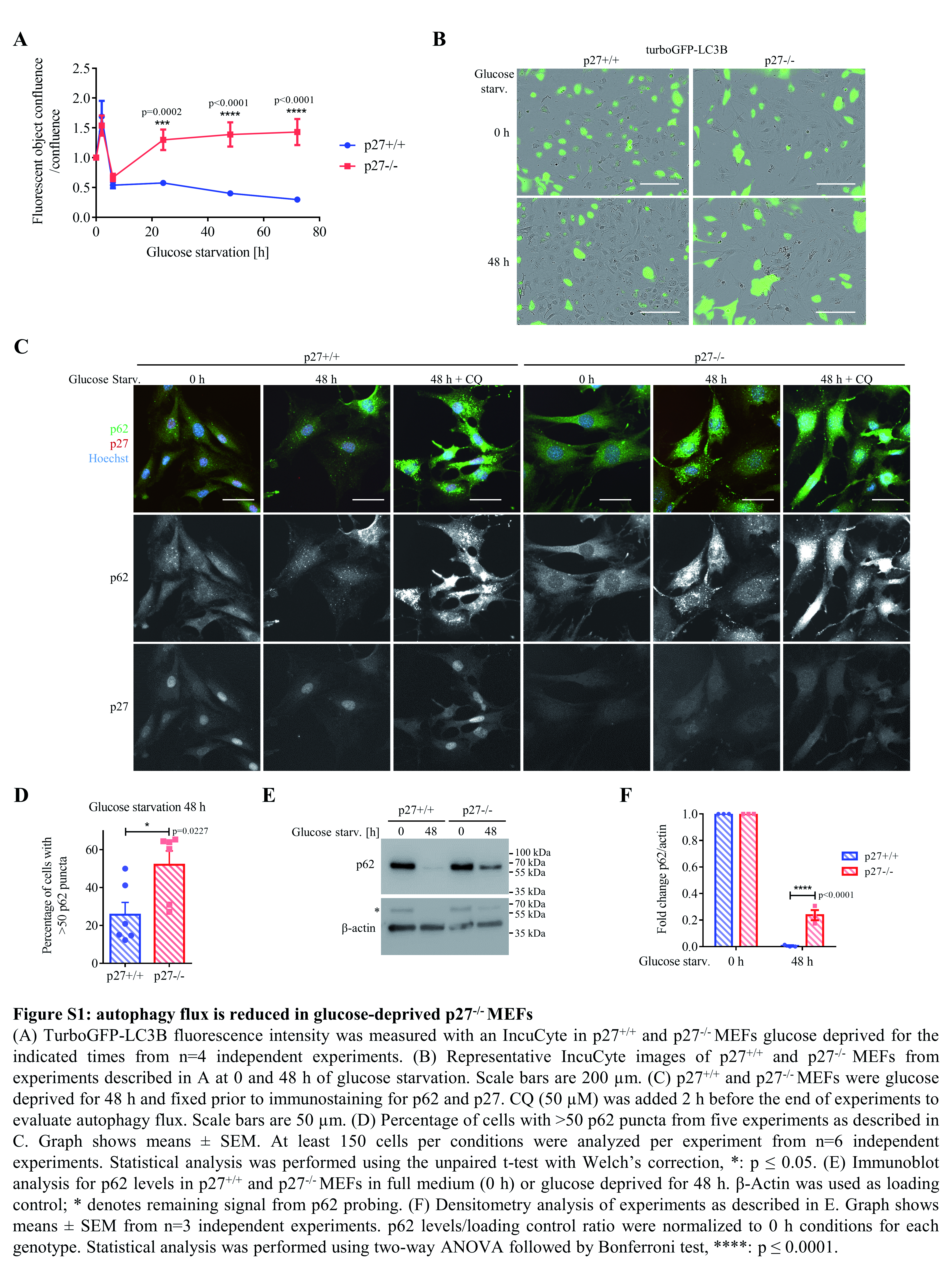

Supplement: Supplementary file 1 — Figure S1 [file 41419_2021_3759_MOESM1_ESM.tif]

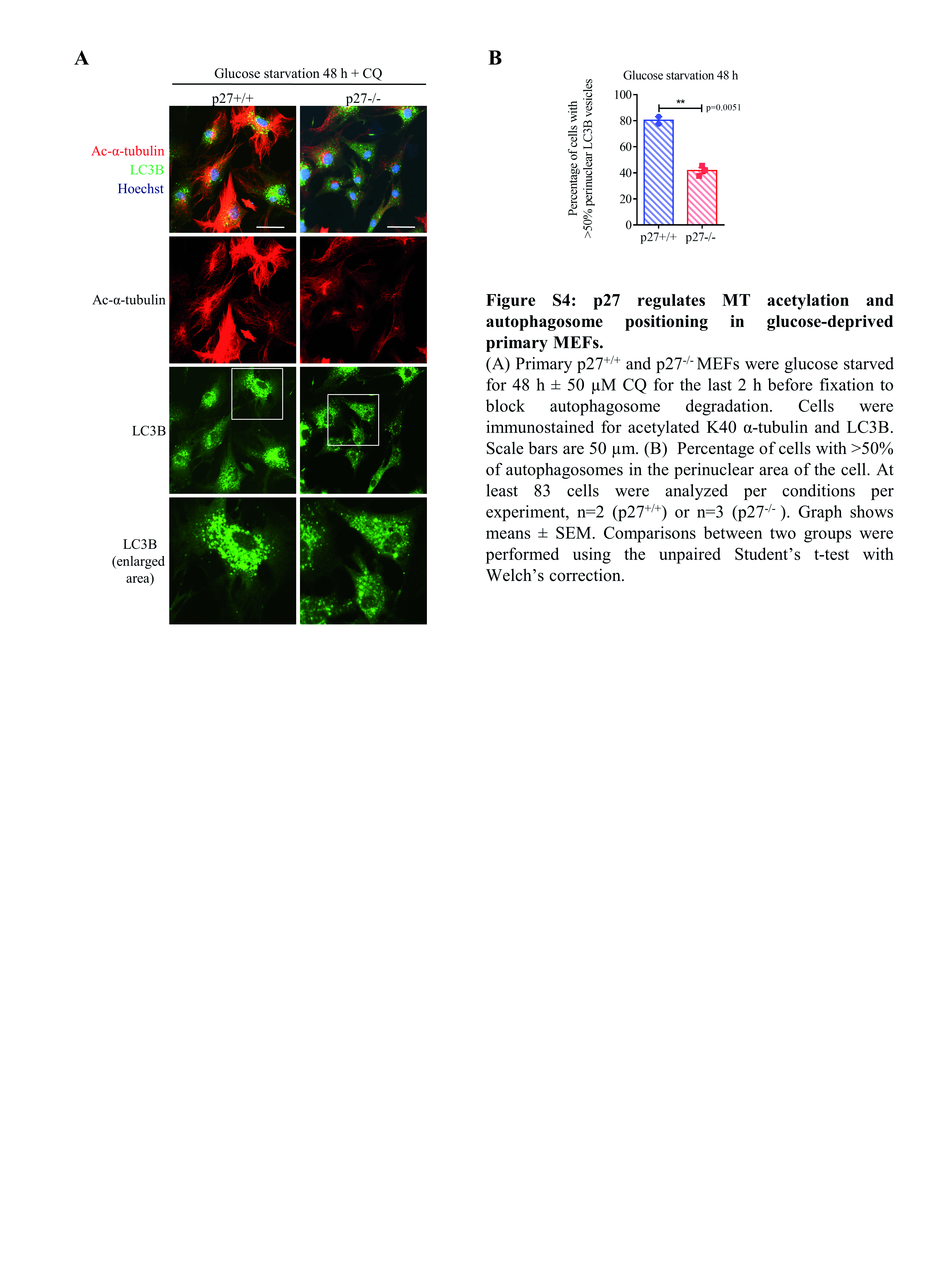

Supplement: Supplementary file 4 — Figure S4 [file 41419_2021_3759_MOESM4_ESM.tif]

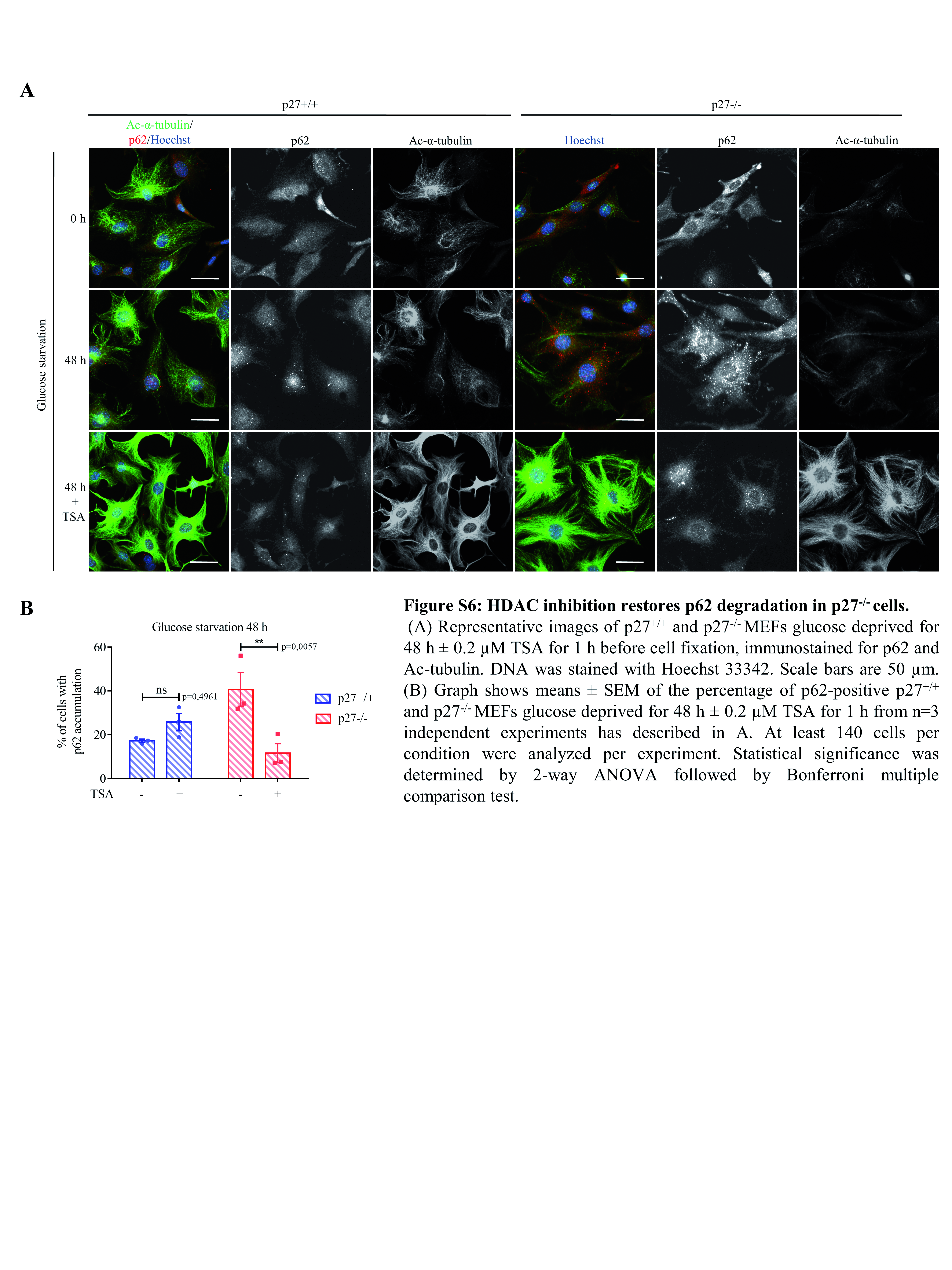

Supplement: Supplementary file 6 — Figure S6 [file 41419_2021_3759_MOESM6_ESM.tif]
